# The Osteogenic Differentiation Effect of the FN Type 10-Peptide Amphiphile on PCL Fiber

**DOI:** 10.3390/ijms19010153

**Published:** 2018-01-04

**Authors:** Ye-Rang Yun, Hae-Won Kim, Jun-Hyeog Jang

**Affiliations:** 1 Industrial Technology Research Group, Research and Development Division, World Institute of Kimchi, Nam-Gu, Gwangju 61755, Korea; yunyerang@wikim.re.kr; 2 Institute of Tissue Regeneration Engineering (ITREN), Dankook University, Cheonan 330-714, Korea; 3 Department of Nanobiomedical Science & BK21 PLUS NBM Global Research Center for Regenerative Medicine, Dankook University, Cheonan 330-714, Korea; 4 Department of Biomaterials Science, School of Dentistry, Dankook University, Cheonan 330-714, Korea; 5 Department of Biochemistry, Inha University School of Medicine, Incheon 22212, Korea

**Keywords:** FNIII10, peptide amphiphile, PCL fiber, osteogenic differentiation activity

## Abstract

The fibronectin type 10-peptide amphiphile (FNIII10-PA) was previously genetically engineered and showed osteogenic differentiation activity on rat bone marrow stem cells (rBMSCs). In this study, we investigated whether FNIII10-PA demonstrated cellular activity on polycaprolactone (PCL) fibers. FNIII10-PA significantly increased protein production and cell adhesion activity on PCL fibers in a dose-dependent manner. In cell proliferation results, there was no effect on cell proliferation activity by FNIII10-PA; however, FNIII10-PA induced the osteogenic differentiation of MC3T3-E1 cells via upregulation of bone sialoprotein (*BSP*), collagen type I (*Col I*), osteocalcin (*OC*), osteopontin (*OPN*), and runt-related transcription factor 2 (*Runx2*) mitochondrial RNA (mRNA) levels; it did not increase the alkaline phosphatase (*ALP*) mRNA level. These results indicate that FNIII10-PA has potential as a new biomaterial for bone tissue engineering applications.

## 1. Introduction

Recently, various attempts have been made to find new biomaterial for tissue engineering. Among the attempts, research on the peptide amphiphile (PA) has been widely reported. PA has hydrophilic and hydrophobic components and a self-assembly ability as a peptide-based molecule. There is increased interest in the extracellular matrix (ECM) molecule mimetic PA because it can promote certain cellular activities including adhesion, proliferation, and differentiation [1,2,3,4,5]. Based on these characteristics, biomimetic PA is extensively utilized for tissue engineering as a new biomaterial. For example, the Arg-Gly-Asp (RGD) peptide, composed of L-arginine, glycine, and L-aspartic acid, participates in cellular attachment via integrin [6]. For this reason, numerous studies on PA-RGD have been reported in the pharmaceutical field [7,8,9] as well as in the tissue engineering field [10,11,12]. For instance, the combination of hydroxyapatite (HA) and PA-RGD enhances the osteoinductive and osteoconductive activities of the scaffold [13].

Moreover, studies on amphiphile containing fibronectin (FN) are commonly demonstrated and reported. We also previously demonstrated that the engineered fibronectin type 10-peptide amphiphile sequence (FNIII10-PA) potentiates utilization for bone tissue engineering by enhancing the adhesion, proliferation, and differentiation of rat bone marrow stem cells (rBMSCs) [14]. In another study, the tenascin-C mimetic PA (TN-C PA) showed self-assembly activity. Nanofiber gels containing TN-C PA were shown to promote neurite outgrowth, which could potentially be used for artificial matrix therapy in neuronal regeneration [15].

Polycaprolactone (PCL) is a biomaterial that is used mostly for bone tissue engineering [16,17]. PCL is a biodegradable polyester with a low melting point (60 °C) and a low glass transition temperature (−60 °C). Because of its biodegradation properties, PCL is regarded as a suitable biomaterial for long-term implantable devices [18]. Above all, PCL has advantages in tissue engineering applications for drug delivery devices, sutures, or adhesion barriers without safety problems for the Food and Drug Administration (FDA) approval [19]. However, it is difficult for PCL to influence cellular activity because it does not provide an ECM type structure for the cells [20]. Recently, nanofibrous PCL has been the preferred choice for bone tissue engineering applications by improving cellular activities and mechanical properties. PCL is indisputably the most used biomaterial in bone tissue engineering research.

Previously, we revealed that genetically engineered FNIII10-PA promoted cell adhesion, proliferation, and differentiation of rBMSCs. In the present study, we investigated whether or not FNIII10-PA with PCL fiber promoted the MC3T3-E1’s cellular activities. FNIII10 was used as the negative control in all experiments. Initially, the protein adhesion activity of FNIII10-PA was measured. Then, the effects of FNIII10-PA with PCL fiber on MC3T3-E1 cells adhesion and proliferation were investigated. Furthermore, the osteogenic differentiation activity of FNIII10-PA with PCL fibers on MC3T3-E1 cells was investigated by measuring the osteogenic differentiation marker mRNA level.

## 2. Results and Discussion

### 2.1. Morphology of PCL Fibers

**Figure 1:**
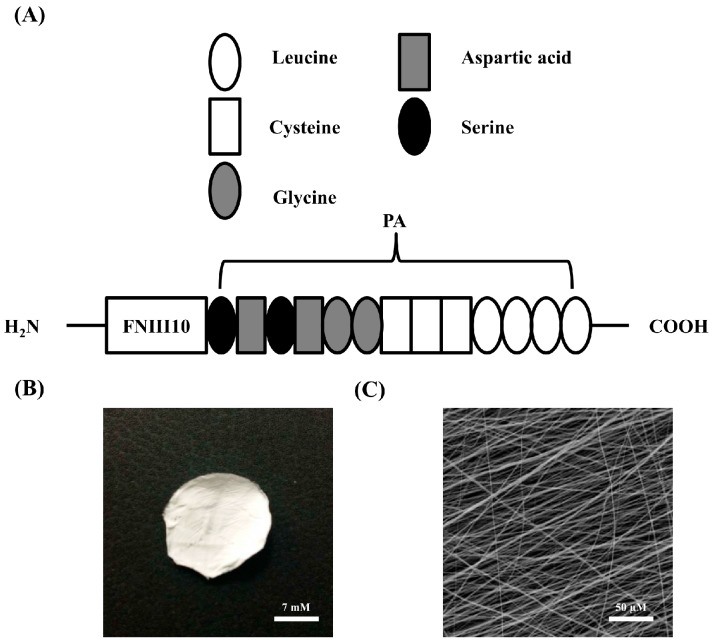
Structure of fibronectin type 10-peptide amphiphile (FNIII10-PA) (**A**) and morphology of polycaprolactone(PCL) fiber (**B**,**C**). Peptide amphiphile (PA) sequence, LLLLCCCGGDS (L, leucine; C, cysteine; G, glycine; D, aspartic acid; S, serine) was connected with fibronectin type III10. Scale is 7 mM in panel B, 50 μM in panel C (resolution 500×).

PCL has been commonly used as a biomaterial in the tissue engineering field because of its non-toxic and biodegradable characteristics. On the other hand, PCL demonstrated a slow degradation rate, poor mechanical properties, and low cell adhesion. Particularly, PCL shows low affinity and stiffness in cells due to poor hydrophilicity, leading to the reduction in cell proliferation, migration, differentiation, and regeneration [20]. However, nanofibrous PCL was reported to improve the cellular activities in recent studies [21,22]. FNIII10 was connected to the PA sequence LLLLCCCGGDSDS (L, leucine; C, cysteine; G, glycine; D, aspartic acid; S, serine) to create FNIII10-A sequence as illustrated in Figure 1A. PCL fiber was fabricated by the electrospinning technique. Figure 1B shows that the electrospun PCL fiber was uniform with straight fibers. Average diameter of PCL fibers was approximately 1 μM (Figure 1C). Hence, PCL fiber was suitable to investigate the cell adhesion activity of FNIII10 in combination with biomaterial in the present study.

### 2.2. Protein Adhesion Activity of FNIII10-PA on PCL Fibers

**Figure 2:**
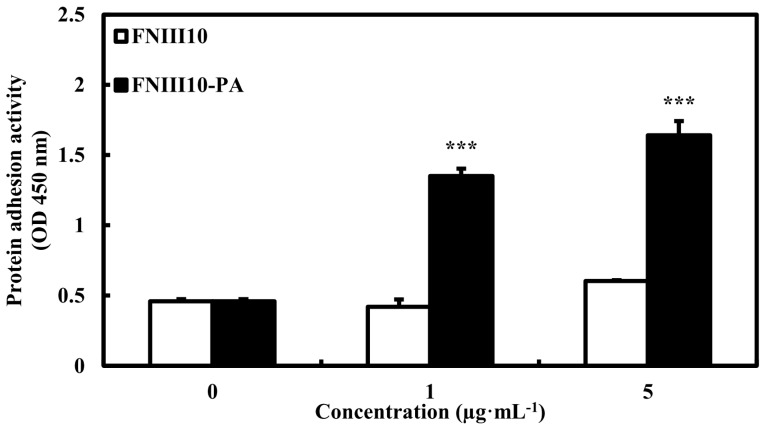
Protein adhesion activity of FNIII10-PA on PCL fiber. PCL fibers were placed in 24-well plates and coated with 0, 1, or 5 μg·mL^−1^ of FNIII10 or FNIII10-PA overnight at 4 °C. Protein adhesion activities are expressed as means ± SD (*n* = 3). *** *p* < 0.001. OD = optical density.

Previously, FNIII10-PA was shown to increase protein adhesion activity on tissue culture plates as compared with FNIII10. As shown in Figure 2, the protein adhesion activity of FNIII10-PA was significantly increased in a dose-dependent manner (*** *p* < 0.001). Particularly, 5 μg·mL^−1^ of FNIII10-PA increased protein adhesion 3-fold compared with that of FNIII10. In many studies, PCL scaffold coated with FN remarkably enhanced cell adhesion, proliferation, and differentiation [23,24,25]. Drevelle et al. found that the PCL film functionalized with RGD peptide (Ac-CGGNGEPRGDTYRAY-NH_2_ derived from the bone sialoprotein) and easily adsorbed the serum adhesive proteins FN and vitronectin (VN), leading to increased cell adhesion and spreading by activating focal adhesion kinase (FAK) signaling [26]. In another study, FN-immobilized nanobioactiveglass (nBG)/PCL (FN-nBG/PCL) scaffolds also increased cell differentiation as well as cell proliferation [27]. In the present study, FNIII10-PA showed higher protein adhesion activity on PCL fibers than that of FNIII10 alone.

### 2.3. Cell Adhesion Activity of FNIII10-PA on PCL Fibers

**Figure 3:**
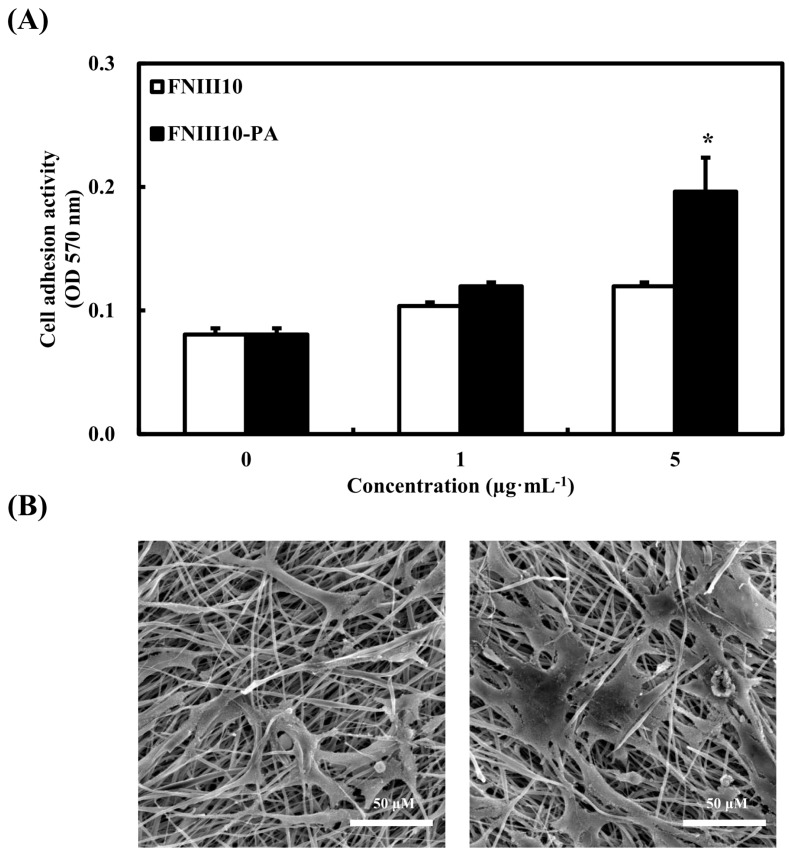
Cell adhesion activity (**A**) and cell spreading (**B**) of MC3T3-E1 on PCL fibers. PCL fibers were placed in 24-well plates and coated with 0, 1, or 5 μg·mL^−1^ of FNIII10 or FNIII10-PA overnight at 4 °C. MC3T3-E1 cells were cultured at a density of 1 × 10^5^ cells and incubated for 30 min at 37 °C. Cell adhesion activities are expressed as means ± SD (*n* = 3). * *p* < 0.05. For SEM analysis, 5 μg·mL^−1^ of FNIII10 and FNIII10-PA were used. Scale is 50 μM (resolution 500×).

As mentioned above, cells are unable to perform many cellular activities on PCL fibers, because PCL fibers do not provide a structure similar to the natural ECM. To evaluate whether or not FNIII10-PA enhances cellular activity on PCL fibers, cell adhesion activity of FNIII10-PA was performed on PCL fibers. In addition, MC3T3-E1 cell spreading on PCL fibers by FNIII10-PA was observed by scanning electron microscopy (SEM). Figure 3A shows that 5 μg·mL^−1^ of FNIII10-PA significantly enhanced cell adhesion activity on PCL fibers (Figure 3A, * *p* < 0.05). Hence, SEM analysis was performed in 5 μg·mL^−1^ of FNIII10 or FNIII10-PA without a treatment group for comparison. In the SEM image, 5 μg·mL^−1^ of FNIII10-PA induced more cell spreading and attachment on PCL fiber compared to that of FNIII10 (Figure 3B). Generally, PCL in the nanofiber form is preferred in bone tissue engineering studies, because PCL shows low cellular activities such as cell adhesion, proliferation, and differentiation. For instance, Yun et al. reported that PCL nanofibers enhanced cellular activities by providing a three-dimensional structure similar to the ECM [28]. In this study, FNIII10-PA enhanced cell adhesion activity on PCL fibers, even though PCL was in the microfiber form. Based on these results, FNIII10-PA may improve the cell interaction with PCL fiber. 

### 2.4. Cell Proliferation Activity of FNIII10-PA on PCL Fiber

**Figure 4:**
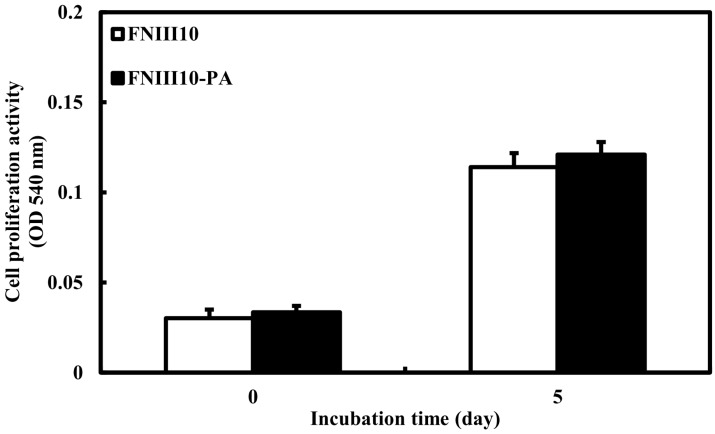
Cell proliferation activity of FNIII10-PA on PCL fibers at 0 and 5 days. PCL fibers were placed in 24-well plates and coated with 5 μg·mL^−1^ of FNIII10 or FNIII10-PA overnight at 4 °C. MC3T3-E1 cells were cultured at a density of 1 × 10^5^ cells and incubated for 5 days at 37 °C. Cell proliferation activity is expressed as mean ± SD (*n* = 3).

We previously revealed that FNIII10-PA was shown to promote rBMSCs proliferation activity. However, MC3T3-El cell proliferation activity of FNIII10-PA on PCL fibers was not observed in the present study. As shown in Figure 4, there were no differences in cell proliferative activity between FNIII10 and FNIII10-PA. Cell proliferative activity of FNIII10-PA could be weak in MC3T3-E1 cells compared to that in rBMSC cells. In the next study, application of FNIII10-PA needs to confirm the rBMSC proliferative activity on PCL fiber. FN was shown to have a powerful effect on both cell adhesion and differentiation of stem cells. For instance, Mohamadyar-Toupkanlou et al. reported that nanofibrous PCL/HA scaffold coated with FN provided a suitable environment for proliferation as well as attachment and differentiation in mouse MSCs (mMSCs) [23]. Shekaran et al. found that ECM-coated PCL microcarriers promoted human early mesenchymal stem cell growth, leading to cell expansion [24]. Similarly, Mousavi et al. revealed that three dimensional nanoscaffold coated with FN induced the human cord blood hematopoietic stem cell expansion [25].

### 2.5. The Osteogenic Differentiation Activity of FNIII10-PA on PCL Fibers

**Figure 5:**
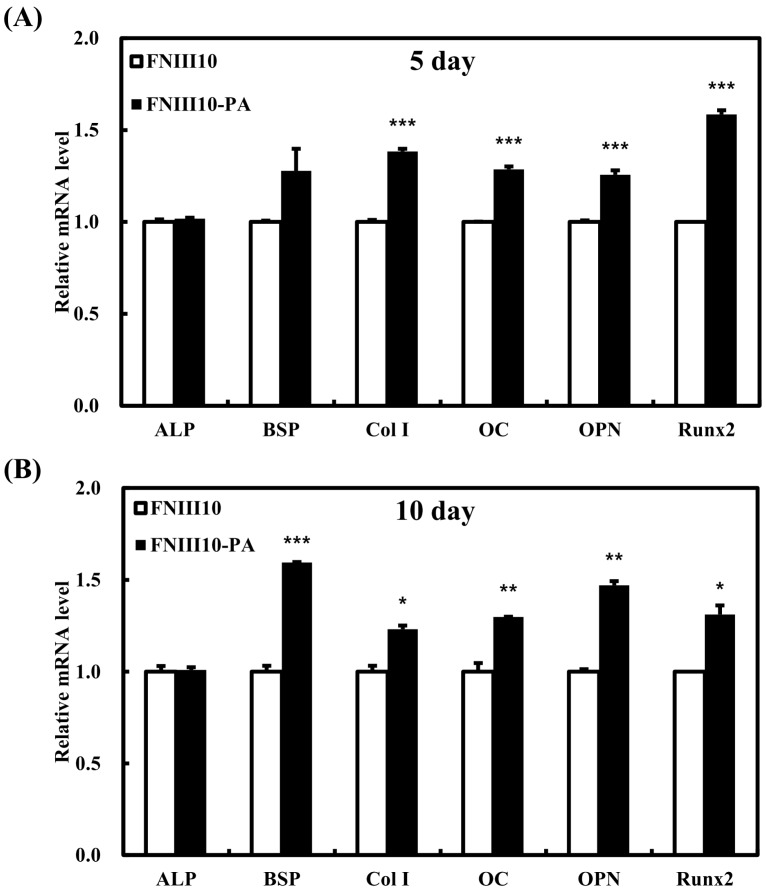
The osteogenic differentiation activity of FNIII10-PA on PCL fibers at 5 days (**A**) and 10 days (**B**). PCL fibers were placed in 24-well plates and coated with 5 μg·mL^−1^ of FNIII10 or FNIII10-PA overnight at 4 °C. MC3T3-E1 cells were cultured at a density of 5 × 10^4^ cells and incubated for 5 and 10 days at 37 °C. Quantitative real-time PCR results were analyzed. * *p* < 0.05, ** *p* < 0.005, and *** *p* < 0.001. Genes: alkaline phosphatase (*ALP)*, bone sialoprotein (*BSP*), collagen type I (*Col I*), osteocalcin (*OC*), osteopontin (*OPN*), and runt-related transcription factor 2 (*Runx2*).

To investigate the osteogenic differentiation activity of FNIII10-PA on PCL fibers, we measured the mitochondrial RNA (mRNA) levels of alkaline phosphatase (*ALP)*, bone sialoprotein (*BSP*), collagen type I (*Col I*), osteocalcin (*OC*), osteopontin (*OPN*), and runt-related transcription factor 2 (*Runx2*) genes at 5 and 10 days. At both time points, the mRNA levels of *BSP*, *Col I*, *OC*, *OPN*, and *Runx2* were significantly upregulated by FNIII10-PA treatment (Figure 5, * *p* < 0.05, ** *p* < 0.005, and *** *p* < 0.001). However, there was no alteration of *ALP* mRNA level at either 5 or 10 days. Interestingly, relative mRNA level of *Col I* was slightly decreased at 10 days compared with that at 5 days. These results were possibly caused by an increase of detached cells. Generally, these markers are expressed with early- or late-stage osteogenic differentiation as follows. *ALP* is a key marker that is responsible for the mineralization of the ECM [29]. *Col I* is the dominant collagen and exists in many tissues such as tendons and ligaments. *ALP* and *Col I* are commonly expressed in the early stage of osteogenic differentiation [30] and are considered early differentiation markers. *OPN* is also an early marker of osteogenic differentiation and is part of non-collagenous bone ECM proteins [31]. *OC* is associated with the late stage of osteogenic differentiation and is known as bone Gla protein (BGP), although *OC* is also a non-collagenous bone ECM protein [32]. As another late differentiation marker, *Runx2* is a master switch for the osteogenic differentiation [33]. In our results, FNIII10-PA significantly induced the osteogenic differentiation of MC3T3-E1 cells on PCL fibers by upregulation of most marker’s mRNA levels at both 5 and 10 days. These results are similar to our previous results [14]. Taken together, we reconfirmed the osteogenic differentiation activity of FNIII10-PA on PCL fibers.

## 3. Materials and Methods

### 3.1. Protein Expression and Purification

After transformation into TOP10 *Escherichia coli*, cells were grown overnight at 37 °C in Luria-Bertani (LB) medium containing ampicillin. Induction was initiated using 0.1% (*w*/*v*) l-arabinose at A600 = 0.6 followed by incubation at 20 °C for 6 h. Bacteria were pelleted by centrifugation at 6000× *g* for 10 min and then lysed and sonicated. A soluble extract was prepared by centrifugation for 30 min at 14,000× *g* in a refrigerated centrifuge, and the supernatant obtained was purified using a nickel-nitrilotriacetic acid resin (Invitrogen, Carlsbad, CA, USA).

### 3.2. Electrospinning of PCL Fibers

A PCL (MW = 80,000; Sigma-Aldrich, St. Louis, MO, USA) solution was prepared by dissolving 10% *w*/*v* PCL in dichloromethane (DCM) and ethanol (4:1 ratio). PCL solutions were ultrasonicated and loaded into a 10 mL plastic syringe equipped with a 21-gauge needle made of stainless steel. The needle was connected to a high-voltage power supply. The tip-to-collector distance was kept at 10 cm. The voltage and injection rate were 15 kV and 0.5 mL·h^−1^, respectively. PCL solution was eletrospun to foil. After drying, PCL fiber (15 mm diameter) was cut from the foil using a punch. All experiments were performed at room temperature (RT). In each experiment, PCL fibers coated with FNIII10 or FNIII10-PA were used.

### 3.3. Morphology of PCL Fibers and Cells on PCL Fibers

The structure of the PCL fibers was observed by SEM at an accelerating voltage of 15 kV after fixing with glutaraldehyde (2.5%), dehydrating with a graded series of ethanol (75%, 90%, 95%, and 100% (*v*/*v*) for 10 min each), treating with hexamethyldisilazane, and coating with platinum.

To observe the cell spreading and expansion on PCL fibers, PCL fibers were placed in 24-well plates and were coated only once with FNIII10 or FNIII10-PA (5 μg·mL^−1^) overnight at 4 °C. Cells were seeded with 1 × 10^5^ cells, incubated for 30 min, washed with Dulbecco’s phosphate-buffered saline (DPBS), the cells on the PCL fibers were fixed with a 3.7% (*w*/*v*) formalin solution for 15 min at RT, dehydrated with a graded series of ethanol (75%, 90%, 95%, and 100% (*v*/*v*) for 10 min each), treated with hexamethyldisilazane, and coated with platinum.

### 3.4. Protein Adhesion Assay

To evaluate the protein adhesion activity, PCL fibers were placed in 24-well plates and were coated only once with FNIII10 or FNIII10-PA (0, 1, or 5 μg·mL^−1^) overnight at 4 °C. Each well was washed with phosphate-buffered saline (PBS) and blocked with 1% (*w*/*v*) bovine serum albumin (BSA) solution for 1 h at RT. After washing with PBS, a peroxidase conjugate of a monoclonal anti-polyhistidine antibody was added and incubated for 1 h at RT. After washing with tris-buffered saline tween (TBS-T), 200 μL Turbo TMB-enzyme-linked immunoserological assay (ELISA) was added and incubated for 30 min at RT. H_2_SO_4_ (100 μL, 2 M) was added to stop the reaction, and the absorbance was read at 450 nm. A protein adhesion assay was performed for the negative control group (un-treated), the positive control group (FNIII10), and the experimental group (FNIII10-PA). The value of the negative control was approximately equal to the value seen with FNIII10 and FNIII10-PA. Hence, results were expressed as the comparison between FNIII10 and FNIII10-PA without negative control.

### 3.5. Cell Culture

MC3T3-E1 is a pre-osteoblastic cell line established from newborn mouse calvarias. We purchased from the American Type Culture Collection (ATCC). Cells were cultured in α-minimum essential media (α-MEM) containing 10% (*v*/*v*) heat-inactivated fetal bovine serum (FBS, WELGENE, Daegu, Korea), 100 units·mL^−1^ penicillin G sodium, 10 μg·mL^−1^ streptomycin, and 25 μg·mL^−1^ amphotericin B (WELGENE, Daegu, Korea) at 37 °C in a humidified atmosphere with 5% CO_2_.

### 3.6. Cell Adhesion Assay

Cell adhesion activity on PCL fibers was measured using the crystal violet assay. PCL fibers were placed in 24-well plates and were coated with FNIII10 or FNIII10-PA (0, 1, or 5 μg·mL^−1^) overnight at 4 °C. Each well was washed with DPBS and blocked with 1% (*w*/*v*) BSA solution for 30 min. Cells were cultured with 1 × 10^5^ cells, incubated for 30 min, washed twice with DPBS, and fixed with 3.7% (*w*/*v*) formalin solution for 15 min at RT. Cells were then stained with 0.25% (*w*/*v*) crystal violet (Sigma, St. Louis, MO, USA) in 2% (*v*/*v*) ethanol/water for 1 h at 37 °C and gently washed three times with DPBS. Cells were then lysed with 2% sodium dodecyl sulfate (SDS) solution and transferred to 96-well plates. Absorbance was read at 570 nm. Results were expressed as the comparison between FNIII10 and FNIII10-PA.

### 3.7. Cell Proliferation Assay

Cell proliferation activity was evaluated using the 3-(4,5-dimethylthiazol-2-yl)-2,5-diphenyltetrazolium bromide (MTT) assay, according to the manufacturer’s instructions (Promega, Madison, WI, USA). FNIII10 and FNIII10-PA protein solutions were coated at 5 μg·mL^−1^ on 24-well plates; cells were cultured with 1 × 10^5^ cells and incubated for 0 and 5 days at 37 °C. Cells were then washed three times with DPBS, and 500 μL of MTT (5 mg·mL^−1^ in PBS) was added to each well. After incubation for 4 h, media were removed, and formazan crystals were dissolved in 200 μL dimethyl sulfoxide (DMSO). Absorbance was read at 540 nm. The cell proliferation assay was performed in the negative control group (un-treated), the positive control group (FNIII10), and the experimental group (FNIII10-PA). After identifying the effect of FNIII10 and FNIII10-PA as compared with that of the negative control, the results were expressed as the comparison between FNIII10 and FNIII10-PA.

### 3.8. Quantitative Real-Time PCR Analysis

Initially, PCL fibers were placed in 24-well plates and were coated with FNIII10 or FNIII10-PA (5 μg·mL^−1^) overnight at 4 °C. Cells were cultured at a density of 5 × 10^4^ cells and incubated for 5 and 10 days at 37 °C. Total RNA was extracted using the Easy-spin RNA Extraction kit (iNtRON, Seoul, Korea), and circular (cDNA) was synthesized. The expression levels of the genes *ALP*, *BSP*, *Col I*, *OC*, *OPN*, *Runx2* were confirmed by quantitative real-time PCR. All real-time PCR analyses were performed using the ABI Step One real-time PCR system. Each reaction was performed in a 20-μL reaction mixture containing 0.1 µM of each primer, 10 µL of 2× SYBR Green PCR master mix (Applied Biosystems, including AmpliTaq Gold DNA polymerase in buffer, a dNTP mix, SYBR Green I dye, Rox dye, and 10 mM MgCl_2_), and 1 µL of template cDNA. The *C*t (cycle threshold) value for each gene was determined using the automated threshold analysis function in the ABI instrument and was normalized with respect to *C*t(GAPDH) to obtain d*C*t (d*C*t = *C*t_(GAPDH)_ − *C*t_(specific gene)_). Finally, the *C*t value of FNIII10-PA was normalized by the *C*t value of FNIII10. The primers used for quantitative real-time PCR are shown in Table 1. Quantitative real-time PCR results were also expressed as a comparison between FNIII10 and FNIII10-PA.

### 3.9. Statistical Analysis

All experiments were conducted in triplicate. Experimental data are expressed as mean ± standard deviation (SD). The Student’s *t*-test with paired data sets was used to determine the significances of differences between FNIII10 and FNIII10-PA. Statistical significance was accepted for *p* values <0.05.

## 4. Conclusions

Previously, we revealed that FNIII10-PA has potential in the osteogenic differentiation activity of rBMSCs. In present study, we also found FNIII10-PA enhanced cell adhesion and osteogenic differentiation activities of MC3T3-E1 cells on PCL fibers. Although the PCL microfiber form had low affinity to cells, the combination with FNIII10-PA significantly enhanced cellular activities including adhesion and differentiation. Taken together, FNIII10-PA potentiates the appropriate utilization for bone tissue engineering with biomaterials as well as working FNIII10-PA alone.

## Figures and Tables

**Table 1:** Sequences of primers used for quantitative real-time PCR.

Genes	Forward Primer	Reverse Primer
*GAPDH*	TCCACTCACGGCAAATTCAAC	AGCCCAAGATGCCCTTCAGT
*ALP*	GGGCAATGAGGTCACATCC	GTCACAATGCCCACGGACTT
*BSP*	CAGAGGAGGCAAGCGTCACT	CTGTCTGGGTGCCAACACTG
*Col I*	GAGGCATAAAGGGTATCGTGG	CATTAGGCGCAGGAAGGTCAGC
*OC*	CATCACTGCCACCCAGAAGAC	CAGTGGATGCAGGGATGATGT
*OPN*	CCAATGAAAGCCATGACCAC	CGACTGTAGGGACGATTGGA
*Runx2*	GGCCGGGAATGATGAGAACTA	GGCCCACAAATCTCAGATCGT

## References

[1] Shin H., Jo S., Mikos A.G. (2003). Biomimetic materials for tissue engineering. Biomaterials.

[2] Goktas M., Cinar G., Orujalipoor I., Ide S., Tekinay A.B., Guler M.O. (2015). Self-assembled peptide amphiphile nanofibers and peg composite hydrogels as tunable ECM mimetic microenvironment. Biomacromolecules.

[3] Anderson J.M., Patterson J.L., Vines J.B., Javed A., Gilbert S.R., Jun H.W. (2011). Biphasic peptide amphiphile nanomatrix embedded with hydroxyapatite nanoparticles for stimulated osteoinductive response. ACS Nano.

[4] Shroff K., Pearce T.R., Kokkoli E. (2012). Enhanced integrin mediated signaling and cell cycle progression on fibronectin mimetic peptide amphiphile monolayers. Langmuir.

[5] Kushwaha M., Anderson J.M., Bosworth C.A., Andukuri A., Minor W.P., Lancaster J.R., Anderson P.G., Brott B.C., Jun H.W. (2010). A nitric oxide releasing, self-assembled peptide amphiphile matrix that mimics native endothelium for coating implantable cardiovascular devices. Biomaterials.

[6] Jeschke B., Meyer J., Jonczyk A., Kessler H., Adamietz P., Meenen N.M., Kantlehner M., Goepfert C., Nies B. (2002). RGD-peptides for tissue engineering of articular cartilage. Biomaterials.

[7] Liu Z., Yu L., Wang X., Zhang X., Liu M., Zeng W. (2016). Integrin (α_v_β_3_) tageted RGD peptide based probe for cancer optical imaging. Curr. Protein Pept. Sci..

[8] Hou J., Diao Y., Li W., Yang Z., Zhang L., Chen Z., Wu Y. (2016). RGD peptide conjugation results in enhanced antitumor activity of PD0325901 against glioblastoma byboth tumor-targeting delivery and combination therapy. Int. J. Pharm..

[9] Song Z., Lin Y., Zhang X., Feng C., Lu Y., Gao Y., Dong C. (2017). Cyclic RGD peptide-modified liposomal drug delivery system for targeted oral apatinib administration: Enhanced cellular uptake and improved therapeutic effects. Int. J. Nanomed..

[10] Chen W., Zhou H., Weir M.D., Tang M., Bao C., Xu H.H. (2013). Human embryonic stem cell-derived mesenchymal stem cell seeding on calcium phosphate cement-chitosan-RGD scaffold for bone repair. Tissue Eng. Part A.

[11] Chen L., Li B., Xiao X., Meng Q., Li W., Yu Q., Bi J., Cheng Y., Qu Z. (2015). Preparation and evaluation of an Arg-Gly-Asp-modified chitosan/hydroxyapatite scaffold for application in bone tissue engineering. Mol. Med. Rep..

[12] Kim H.D., Heo J., Hwang Y., Kwak S.Y., Park O.K., Kim H., Varghese S., Hwang N.S. (2015). Extracellular-matrix-based and Arg-Gly-Asp-modified photopolymerizing hydrogels for cartilage tissue engineering. Tissue Eng. Part A.

[13] Çakmak S., Çakmak A.S., Gümüşderelioğlu M. (2013). RGD-bearing peptide-amphiphile-hydroxyapatite nanocomposite bone scaffold: An in vitro study. Biomed. Mater..

[14] Yun Y.R., Pham L.B.H., Yoo Y.R., Lee S., Kim H.W., Jang J.H. (2015). Engineering of self-assembled fibronectin matrix protein and its effects on mesenchymal stem cells. Int. J. Mol. Sci..

[15] Berns E.J., Álvarez Z., Goldberger J.E., Boekhoven J., Kessler J.A., Kuhn H.G., Stupp S.I. (2016). A tenascin-C mimetic peptide amphiphile nanofiber gel promotes neurite outgrowth and cell migration of neurosphere-derived cells. Acta Biomater..

[16] Gómez-Lizárraga K.K., Flores-Morales C., Del Prado-Audelo M.L., Álvarez-Pérez M.A., Piña-Barba M.C., Escobedo C. (2017). Polycaprolactone- and polycaprolactone/ceramic-based 3D-bioplotted porous scaffolds for bone regeneration: A comparative study. Mater. Sci. Eng. C Mater. Biol. Appl..

[17] Xu T., Miszuk J.M., Zhao Y., Sun H., Fong H. (2015). Electrospun polycaprolactone 3D nanofibrous scaffold with interconnected and hierarchically structured pores for bone tissue engineering. Adv. Healthc. Mater..

[18] Dash T.K., Konkimalla V.B. (2012). Poly-ε-caprolactone based formulations for drug delivery and tissue engineering: A review. J. Control. Release.

[19] Li Z., Tan B.H. (2014). Towards the development of polycaprolactone based amphiphilic block copolymers: Molecular design, self-assembly and biomedical applications. Mater. Sci. Eng. C Mater. Biol. Appl..

[20] Ghasemi-Mobarakeh L., Prabhakaran M.P., Morshed M., Nasr-Esfahani M.H., Ramakrishna S. (2008). Electrospun poly(ε-caprolactone)/gelatin nanofibrous scaffolds for nerve tissue engineering. Biomaterials.

[21] Kim M., Kim G. (2012). Electrospun PCL/phlorotannin nanofibres for tissue engineering: Physical properties and cellular activities. Carbohydr. Polym..

[22] Chen J.P., Chang Y.S. (2011). Preparation and characterization of composite nanofibers of polycaprolactone and nanohydroxyapatite for osteogenic differentiation of mesenchymal stem cells. Colloids Surf. B Biointerfaces.

[23] Mohamadyar-Toupkanlou F., Vasheghani-Farahani E., Hanaee-Ahvaz H., Soleimani M., Dodel M., Havasi P., Ardeshirylajimi A., Taherzadeh E.S. (2017). Osteogenic differentiation of MSCs on fibronectin-coated and nHA-modified scaffolds. ASAIO J..

[24] Shekaran A., Lam A., Sim E., Jialing L., Jian L., Wen J.T., Chan J.K., Choolani M., Reuveny S., Birch W. (2016). Biodegradable ECM-coated PCL microcarriers support scalable human early MSC expansion and in vivo bone formation. Cytotherapy.

[25] Mousavi S.H., Abroun S., Soleimani M., Mowla S.J. (2015). Expansion of human cord blood hematopoietic stem/progenitor cells in three-dimensional Nanoscaffold coated with Fibronectin. Int. J. Hematol. Oncol. Stem Cell Res..

[26] Drevelle O., Bergeron E., Senta H., Lauzon M.A., Roux S., Grenier G., Faucheux N. (2010). Effect of functionalized polycaprolactone on the behaviour of murine preosteoblasts. Biomaterials.

[27] Won J.E., Mateos-Timoneda M.A., Castano O., Planell J.A., Seo S.J., Lee E.J., Han C.M., Kim H.W. (2015). Fibronectin immobilization on to robotic-dispensed nanobioactive glass/polycaprolactone scaffolds for bone tissue engineering. Biotechnol. Lett..

[28] Yun Y.P., Kim S.J., Lim Y.M., Park K., Kim H.J., Jeong S.I., Kim S.E., Song H.R. (2014). The effect of alendronate-loaded polycarprolactone nanofibrous scaffolds on osteogenic differentiation of adipose-derived stem cells in bone tissue regeneration. J. Biomed. Nanotechnol..

[29] Marom R., Shur I., Solomon R., Benayahu D. (2005). Characterization of adhesion and differentiation markers of osteogenic marrow stromal cells. J. Cell. Physiol..

[30] Tsai M.T., Li W.J., Tuan R.S., Chang W.H. (2009). Modulation of osteogenesis in human mesenchymal stem cells by specific pulsed electromagnetic field stimulation. J. Orthop. Res..

[31] Zohar R., Cheifetz S., McCulloch C.A., Sodek J. (1998). Analysis of intracellular osteopontin as a marker of osteoblastic cell differentiation and mesenchymal cell migration. Eur. J. Oral Sci..

[32] Candeliere G.A., Liu F., Aubin J.E. (2001). Individual osteoblasts in the developing calvaria express different gene repertoires. Bone.

[33] Li S., Kong H., Yao N., Yu Q., Wang P., Lin Y., Wang J., Kuang R., Zhao X., Xu J. (2011). The role of runt-related transcription factor 2 (Runx2) in the late stage of odontoblast differentiation and dentin formation. Biochem. Biophys. Res. Commun..

